# Pharmacokinetics of Marine-Derived Drugs

**DOI:** 10.3390/md18110557

**Published:** 2020-11-09

**Authors:** Alexander N. Shikov, Elena V. Flisyuk, Ekaterina D. Obluchinskaya, Olga N. Pozharitskaya

**Affiliations:** 1 Department of Technology of Pharmacutical Formulations, St. Petersburg State Chemical Pharmaceutical University, Prof. Popov, 14a, Saint-Petersburg 197376, Russia; elena.flisyuk@pharminnotech.com; 2 Murmansk Marine Biological Institute of the Russian Academy of Sciences (MMBI RAS), Vladimirskaya, 17, Murmansk 183010, Russia; oluchinskaya@mmbi.info (E.D.O.); pozharitskaya@mmbi.info (O.N.P.)

**Keywords:** aplidine, astaxanthin, dolastatin, echinochrome, echinoside, fucoidan, fucoxanthin, halomon, holothurin, ilimaquinone

## Abstract

Marine organisms represent an excellent source of innovative compounds that have the potential for the development of new drugs. The pharmacokinetics of marine drugs has attracted increasing interest in recent decades due to its effective and potential contribution to the selection of rational dosage recommendations and the optimal use of the therapeutic arsenal. In general, pharmacokinetics studies how drugs change after administration via the processes of absorption, distribution, metabolism, and excretion (ADME). This review provides a summary of the pharmacokinetics studies of marine-derived active compounds, with a particular focus on their ADME. The pharmacokinetics of compounds derived from algae, crustaceans, sea cucumber, fungus, sea urchins, sponges, mollusks, tunicate, and bryozoan is discussed, and the pharmacokinetics data in human experiments are analyzed. In-depth characterization using pharmacokinetics is useful for obtaining information for understanding the molecular basis of pharmacological activity, for correct doses and treatment schemes selection, and for more effective drug application. Thus, an increase in pharmacokinetic research on marine-derived compounds is expected in the near future.

## 1. Introduction

Marine organisms represent an excellent source of innovative compounds, having tremendous potential for the development of new drugs. Although marine species are used in traditional medicines in different countries [1,2,3,4,5,6], a limited number of marine-derived drugs (such as fish oil, glucosamine, and protamine sulfate) have been used in officinal medicine for a long time [7,8,9]. Recent years have been marked by rapid growth in the discovery of new marine-derived molecules and in the study of their pharmacological activity. About 1200–1500 new compounds are annually reported in the literature [10,11,12,13,14]. In the last decades, unique compounds that show a broad spectrum of biological activities have been isolated from marine organisms: antiallergic, anti-atherosclerotic, antibacterial, anticancer, anticoagulant, antidiabetic, antifungal, antihyperlipidemic, antihypertensive, anti-inflammatory, antioxidant, antiviral, cardioprotective, immunoadjuvant, hypocholesterolemic, and so on [15,16,17,18,19,20,21,22,23]. However, a relatively low number of active molecules have been subject to extensive pre-clinical studies, and pharmacokinetic data have been provided for the limited number of candidates. In vivo pharmacokinetic studies are complicated; they require investment, a large number of analyses, and the development of highly sensitive and specific analytical methods. It is important to have reliable and validated methodologies that are able to assess the pharmacokinetics of molecule candidates before proceeding to drug formulation development. 

The pharmacokinetics of marine drugs has attracted increasing interest in recent decades due to its effective and potential contribution to the selection of rational dosage recommendations and the optimal use of the therapeutic arsenal. In general, pharmacokinetics studies how a drug changes after administration via the processes of absorption, distribution, metabolism, and excretion (ADME) [24].

Although ADME processes are roughly separated, they are highly integrated phenomena [25]. The key parameters that provide insights into how the drug concentration changes in an organism in course of ADME include the apparent half-life of elimination (T_1/2_), the area under the curve (AUC), clearance (Cl), maximum concentration (C_max_) and time at which C_max_ is observed (T_max_), median residual time (MRT), the high volume of distribution in the blood (V_ss_), and bioavailability (F). Distribution half-life (t_1/2 α_) and elimination half-life (t_1/2 β_) are useful for two-compartment models [26].

This review provides a concise summary of the pharmacokinetic studies of marine-derived active compounds, with a particular focus on their ADME.

## 2. Analytical Methods Used in Pharmacokinetic Studies

Different analytical methods are implemented for the evaluation of pharmacokinetic parameters. The methods most commonly used for the analysis of marine-derived compounds in biomaterials are presented in the Table 1. For the direct analysis of active molecules, these methods include fluorescein labeling, radioactive labeling (total radioactivity analysis (TRA) and radioimmunoassay (RIA)), matrix-assisted laser desorption/ionization (MALDI), MALDI imaging mass spectrometry (IMS), high-performance liquid chromatography (HPLC) or liquid chromatography (LC) with evaporative light-scattering detection (ELSD), fluorescent detection, different mass spectrometry (MS), electrospray ionization (ESI), or UV detection. The other group of methods is based on the enzyme-linked immunosorbent assay (ELISA) and the biomarker approach. The analysis of active compounds in biomaterials requires high sensitivity. The sensitivity depends on the nature of the active compound and the biomaterial. The pharmacokinetics of some compounds, such as fucoidan or aplidine, has been studied using different methods, showing that biomarker-based assays provide high sensitivity and broad linearity (Table 1). 

## 3. Pharmacokinetics Studies in Animals

Although the number of reports about new marine-derived compounds has dramatically increased, only a few of the most promising candidates have been submitted for pharmacokinetic research. Several molecules initially discovered from marine sources were later synthetically modified or simplified. These compounds will be not discussed in this review. 

Seaweeds are rich sources of biologically active phenolics [50]. However, most of them are abundant in terrestrial organisms, and the pharmacokinetics of these compounds is well-described elsewhere. We discuss the absorption, distribution, metabolism, and excretion of compounds that were isolated from marine organisms and those identical to natural compounds. The main pharmacokinetic parameters of some marine-derived compounds are summarized in Table 2.

### 3.1. Absorption

#### 3.1.1. Algal-Derived Compounds

**Figure 1:**
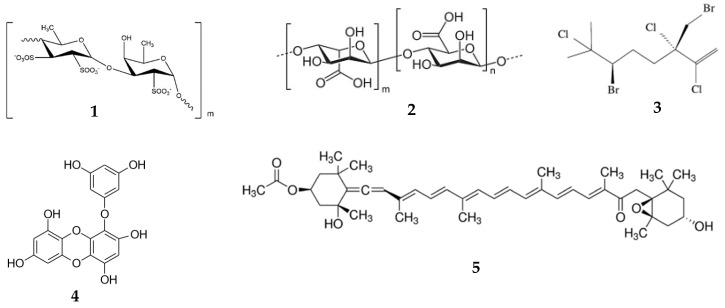

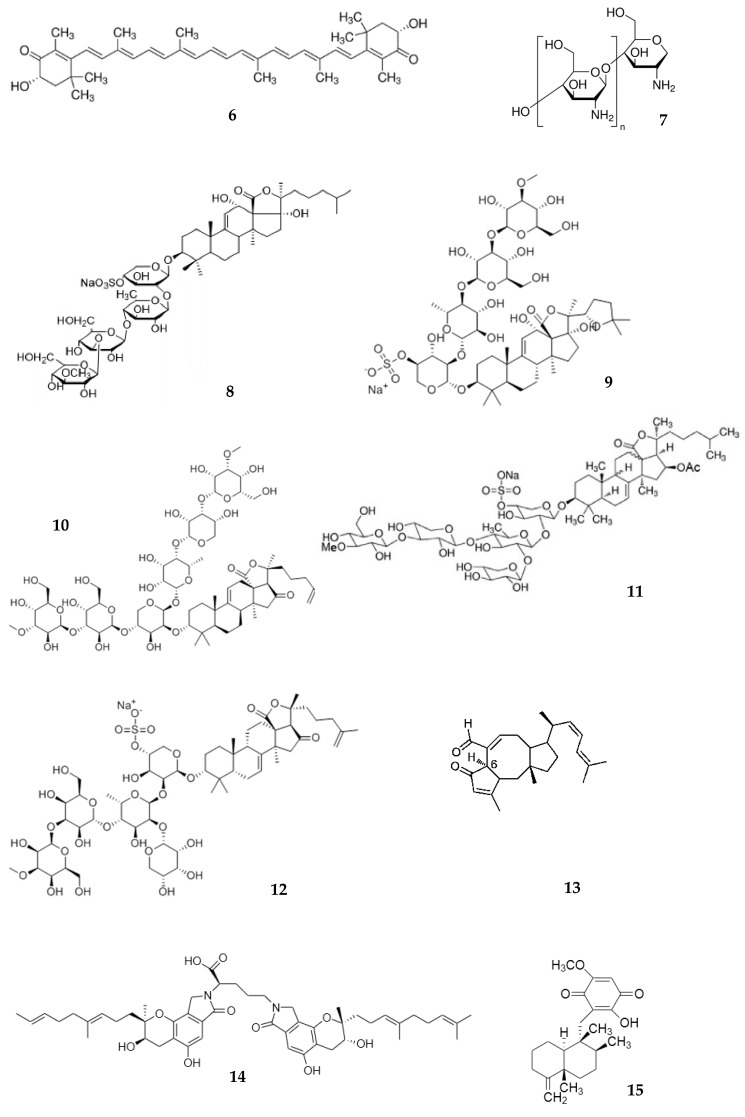

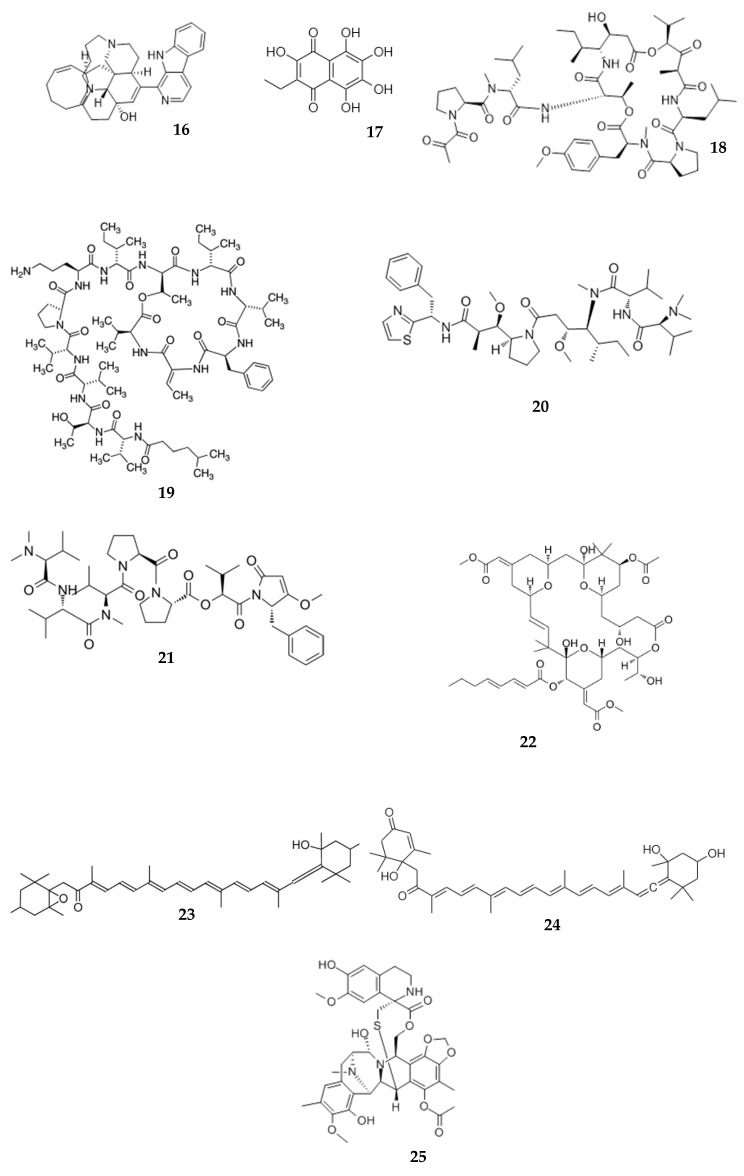
Structures of marine-derived compounds: fucoidan (**1**); alginic acid (**2**); halomon (**3**); eckol (**4**); fucoxanthin (**5**); astaxanthin (**6**); chitosan (**7**); echinoside A (**8**); holothurin A (**9**); holotoxin A1 (**10**); frondoside A (**11**); cucumarioside A_2_-2 (**12**); 6-epi-ophiobolin G (**13**); diindolinonepyrane (**14**); ilimaquinone (**15**); manzamine A (**16**); echinochrome A (**17**); aplidine (**18**); kahalalide F (**19**); dolastatin 10 (**20**); dolastatin 15 (**21**); bryostatin 1 (**22**); fucoxanthinol (**23**); amarouciaxanthin A (**24**); ecteinascidin 743 (**25**).

Fucoidan (Figure 1, (**1**)) is a fucose-rich sulfated heteropolysaccharide found in various algae. It shows a broad spectrum of pharmacological activities including anticancer, anti-coagulant, anti-diabetic, anti-inflammatory, anti-viral, immunoadjuvant, treatment of renal diseases activities [20,21,34,61,62,63,64,65,66]. The pharmacokinetics of fucoidans from different sources has been reported in rabbits, rats, and mice and after different routes of administration. A low molecular weight (MW) of fucoidan (7.1 kDa) from *Laminaria japonica* rapidly appears in the plasma (C_max_ = 110.53 μg/mL; T_max_ = 5 min) after intravenous (i/v) injection in rabbits (50 mg/kg). The serum concentration–time function was two-exponential with t_1/2 α_ = 11.24 ± 2.93 min and t_1/2 β_ = 98.20 ± 25.78 min. Due to the low sensitivity of the analytical method, fucoidan was only detected at 2 h, but not quantified in the serum of rabbits after peroral administration (200 mg/kg) [36]. The pharmacokinetics of fluorescein isothiocyanate (FITC)-labeled commercially-available fucoidan (MW = 107.8 kDa) from *Fucus vesiculosus* was investigated after i/v injection (50 mg/kg) in mice. The C_max_ in the blood was 66.37 μg/mg, and AUC was 198.11 μg/g·h [32]. The pharmacokinetics of another fucoidan (MW = 100 kDa) from *L. japonica* was reported in rats after i/v injection (6 mg/kg) and peroral administration (20 mg/kg). The C_max_ was 75.59 μg/mL, and AUC was 479.07 g·h/mL after i/v injection. C_max_ was 7.33 μg/mL, T_max_ was 2 h, and AUC was 42.69 g·h/mL after peroral administration [35]. Low- (7.6 kDa) and medium-MW (35 kDa) fucoidans from *L. japonica* were administered perorally to rats at doses of 200, 400, and 800 mg/kg. The absorption of medium-MW fucoidan was lower than the absorption of low-MW fucoidan. The C_max_ levels of fucoidan in blood after administration of 800 mg/kg were 151.7 μg/mL for low MW at T_max_ = 15 h and 131.81 μg/mL for medium MW at T_max_ = 25 h [34]. Recently, the pharmacokinetics of fucoidan (MW = 735 kDa) from *Fucus vesiculosus* was investigated in rats after peroral administration, i/v injection, and transdermal application at the dose of 100 mg/kg. After i/v injection, C_max_ was 9.15 ± 0.60 µg/mL [28]. Fucoidan was detected in plasma 30 min after peroral administration with C_max_ of 0.125 µg/mL and T_max_ was 4 h [38], whereas after transdermal application of ointment, C_max_ was 0.12 µg/mL at 1.2 h [28]. Nagamine et al. [40] demonstrated that fucoidan from *Cladosiphon okamuraus* (MW = 56 kDa) penetrates across the Caco-2 cell monolayer by active transport with maximal activity at one hour, followed by a rapid decrease. Later, the clathrin endocytic pathway was established to be involved in the absorption and transport of fucoidan [32]. All these results proved that fucoidan rapidly absorbs after different routes of administration.

Griffithsin (GRFT) is a homodimeric lectin containing 121 amino acids, with a total of six oligosaccharide binding sites, and with terminal oligomannose residues on asparagine (N)-linked Man5-9-GlcNAc2 structures. It has been isolated from the red algae *Griffithsia* spp. [67]. GRFT shows promising anti-viral activity [67,68,69]. GRFT was administered to rats i/v (10–20 mg/kg), perorally (10 mg/mL), and subcutaneously (s/c; 10 or 20 mg/kg). Additionally, the pharmacokinetics of GRDT was studied after 10 days peroral administration at the dose of 20 or 40 mg/kg. The concertation of GRFT in serum after i/v injection was dose dependent, with mean concentrations of 74 and 141 µg/mL at 10 and 20 mg/kg, respectively, and then rapidly decreased. The plasma concentration–time function was two-exponential with mean t_1/2α_ of 0.5 h and t_1/2β_ of 1.9 h, without statistically significant dose dependence. After s/c injection, GRFT appeared in serum at 15 min and gradually increased up to T_max_ of 4 h, which suggests its slow absorption. The mean absorption t_1/2α_ was 1.45 h and mean distribution t_1/2β_ was 2.45 h, without statistically significant dose dependence. The C_max_ after s/c injection was 12-fold lower compared with i/v injection and was dose dependent. The difference in AUC was 2.4-fold only at 10 mg/kg and was statistically not significant at 20 mg/kg (Table 2). GRFT was not detected in serum in the 96 h after a single dose (20 mg/kg) of peroral administration [51]. The C_max_ level of GRFT was 0.32 and 0.46 µg/mL in plasma of mice and guinea pigs, respectively, observed by day 11 after 14 days’ consecutive s/c injection (10 mg/kg) [68]. 

Alginates (**2**) represent a group of copolymers of mannuronic and guluronic acids. These anionic polysaccharides are isolated predominantly from brown algae. Due to their high ability for gel formation and their specific viscosity-related properties, alginates have been extensively used in pharmaceutical formulations as hydrophilic drug carriers, for coating, film formation, and as matrix materials for targeted drug delivery [70,71,72]. Two sodium alginates (MW = 200 kDa) with uluronic acid contents of about 44% and < 5% were not absorbed following peroral administration to mice. After i/v injection, the plasma concentration–time function was two-exponential with t_1/2α_ of 4 h min and t_1/2β_ of 22 h. After i/p injection, the C_max_ in plasma was observed at 5–6 h [73]. Alginate oligosaccharide (AOs), containing the dimer, trimer, and tetramer of commercial sodium alginate, was obtained by lyase enzyme degradation. In contrast to the data reported by Hagen et al., [73] AOs was detected in the plasma of mice after peroral administration (10 mg per mice) with C_max_ of 24.5 µg/mL (about 0.49% of administered dose) at T_max_ of 5 min, followed by a rapid decrease. After 2 h, AOs was below the detection limit [43].

Halomon (**3**), a halogenated monoterpene 6(*R*)-bromo-3(*S*)-(bromomethyl)-7-methyl-2,3,7-trichloro-1-octene, was isolated from the red alga *Portieria hornemannii*. It demonstrates potent activity against cancer cells [74,75]. Pharmacokinetics of halomon was studied in mice after i/v (20–135 mg/kg), i/p, s/c, and peroral (135 mg/kg) administrations. The concentration of halomon in plasma at 5 min after i/v injection was 85–100 µg/mL for female and 65–67 µg/mL for male mice. The pharmacokinetics of halomon in plasma was described by a two-compartment open linear model and was linear at all doses (20–135 mg/kg), as evidenced by the peak concentration and AUC. The bioavailability after s/c injection was 45%; after peroral administration, it was 4% [33].

Eckol (**4**) belongs to phlorotannins, which are derivatives of dibenzo-1,4-dioxins. It contains phloroglucinol components linked to each other. Red and brown algae, in particular *Ecklonia stolonifera*, are known to produce eckol. Its therapeutic potential includes, but is not limited to, anti-bacterial anti-cancer, ant-coagulant, anti-diabetic, anti-hypertensive, and neuroprotective, radioprotective activities [76]. The pharmacokinetics of eckol was analyzed in silico using PreADMET software. Eckol was considered the lipophilic compound with logPo/w = 2.99. The human intestinal absorption was calculated as moderate (55.60%), and the plasma protein binding of eckol was 100%. The predicted in vivo blood–brain barrier penetration indicated moderate absorption by the central nervous system [77].

Fucoxanthin (**5**) is an abundant carotenoid present in algae and diatoms. It shows multifunctional biological activity and has been used as a valuable nutrient [78,79]. The pharmacokinetics of fucoxanthin isolated from *L. japonica* was studied in mice after peroral administration (160 nmol per mice). At one hour after administration, metabolites fucoxanthinol and amarouciaxanthin A were detected in the plasma of mice. Although the T_max_ for both metabolites was 4 h, the C_max_ for fucoxanthinol was about twice as high compared with amarouciaxanthin A. The difference in the AUC between these two metabolites was approximately 1.4-fold [80]. Fucoxanthin (2 mg/kg) was quickly metabolized to fucoxanthinol, which was detected 5 min post-injection in rats. The AUC for fucoxanthin was about 2.5-fold higher when compared with fucoxanthinol. After peroral administration (65 mg/kg), the absorption rate was much slower. The same quick conversion of fucoxanthin to fucoxanthinol was noted. Both compounds were detected in plasma 0.5 h post-administration. The T_max_ for fucoxanthin was 7.7 h vs. 11 h for fucoxanthinol (Table 2) [42]. 

Astaxanthin (**6**) (3,3′-dihydroxy-β,β-carotene4,4′-dione) is a common pigment existing in some bacteria, crustaceans (shrimps, lobsters), and fish (salmon), yet microalgae and phytoplankton are the most abundant sources of astaxanthin [81,82]. It shows multiple pharmacological effects including anti-cancer [83], anti-diabetic [21], geroprotective [84], and neuroprotective [85] effects, and shows use for the treatment of ocular diseases [86]. Commercially available astaxanthin was i/v and perorally administered to rats. Differences were observed in astaxanthin pharmacokinetics after i/v injection (5–20 mg/kg) and peroral administration (100 and 200 mg/kg). The AUC after i/v injection at 20 mg/kg was y 55% greater than at 5 mg/kg. The C_max_ after peroral administration at 200 mg/kg was higher than that at 100 mg/kg, but the difference, as well as the difference in T_max_, were statistically not significant. The AUC after peroral administration at 200 mg/kg was about twice that at 100 mg/kg. The AUC of astaxanthin in plasma after peroral administration was 90.1% lower than after i/v injection to the portal vein of rats (Table 2) [52].

#### 3.1.2. Crustacean-Derived Compounds

Chitosan (**7**) is a cationic polysaccharide, a copolymer of *N*-acetyl-d-glucosamine and β-1–4-linked d-glucosamine, which is obtained by the deacetylation of chitin. The core source of chitosan is the exoskeleton of crustaceans. Due to its unique properties, chitosan is extensively used in pharmaceutical formulations and biomedicine [87,88,89]. Beside this, chitosan is reported to be useful for body weight reduction, in the treatment of neuronal disorders, and for its anti-bacterial, antihypertensive, etc. activities [90,91,92]. The commercial ^125^I-labelled chitosan (MW = 5–10 kDa) was found in blood at 5 min after i/v injection in rats [93]. The pharmacokinetics of commercial water-soluble chitosan with MW of 3.8–230 kDa was investigated in vitro and in vivo. In vitro, chitosan penetrates across the Caco-2 cell layer in a dose-dependent manner. The highest permeability was observed for the low-MW (3.8 kDa) form. The penetration rate decreased with an MW increase. Similar results were observed in rats. After the peroral administration of chitosan (20 mg/kg), C_max_ in plasma ranged between 20.23 and 4.32 μg/mL for low MW and high MW at a T_max_ of 30 min. AUC was dose dependent between 24.13 and 0.97 μg/mL·h. The absorption of chitosan with MW of 3.8 kDa was 25-fold higher compared with an MW of 230 kDa [53]. Similar results were obtained in another study. Chitosans with MW of 990 Da to 76 kDa were prepared from commercial chitosan and administered perorally in mice. The best absorption (C_max_ 0.68 mg/g, T_max_ = 30 min in plasma) was observed for chitosan with an MW of 990 Da. The C_max_ for all other chitosan with higher MW was lower and was observed at T_max_ of 1 h. The result indicated that the intestinal absorption of chitosan increases with decreasing molecular weight. The result also confirmed that the intestinal absorption of chitosan with lower MW is more rapid [54].

#### 3.1.3. Sea Cucumber-Derived Compounds

Sea cucumbers are echinoderms belonging to the Holothuriidae. These organisms have been used as functional food [4] and a source of bioactive compounds [94]. The extract, containing lanostane-type triterpene oligoglycosides echinoside A (**8**) (EA) and holothurin A (**9**) (HA), was isolated from sea cucumber *Pearsonothuria graeffei*. Among others, EA and HA show promising anticancer activity [95,96]. The pharmacokinetic was characterized after single i/v (0.2 mg/kg) and peroral (30 mg/kg) administration of extract to rats. Both saponins were rapidly absorbed and appeared in serum at 4.0 (EA) and 0.94 μg/mL (HA) in the first minute after i/v injection. The serum concentration–time functions for EA and HA are characterized with two maxima after peroral administration. The concentrations of EA and HA at 0.5 h reached 0.11 and 0.2 µg/mL, respectively. The first C_max_ was 0.83 µg/mL (EA) and 0.34 µg/mL (HA) at a T_max_ of 3 h and the second C_max_ were 0.24 µg/mL (T_max_ = 7 h) for EA and 0.27 µg/mL (T_max_ = 9 h) for HA [39]. 

Li et al. investigated echinoside A (EA) (**8**) and holotoxin A1 (HA1) (**10**), isolated from *Apostichopus japonicas* [38]. The cytotoxic and radioprotective effects have been described, among others, for HA1 [97,98]. EA and HA1 (20 mg/kg) were i/v and perorally administered to rats. Both saponins were quickly detected in plasma after i/v injection with C_max_ of 4.0 and 2.87 µg/mL for EA and HA1, respectively. However, the AUC value for EA was 2.5-fold higher than for HA1. After oral administration, EA was detected in rat plasma 1 h, followed by an increase up to 3 h (Table 2). This is evidence of its gradual absorption into plasma. In contrast, HA1 was not detected in plasma after peroral administration. In vitro, EA penetrates across the Caco-2 cell monolayer by passive diffusion without assistance by P-glycoprotein, whereas the transport of HA1 through the monolayer was poor. This agrees with in vivo data [38]. 

The frondosides A, B, and C represent a group of triterpenoid glycosides isolated from Atlantic sea cucumber *Cucumaria frondosa*. Frondosides are known as potent anticancer compounds against the adenocarcinomas of different organs, as well as leukemia [99,100,101]. The pharmacokinetics of the most active frondoside, frondoside A (**11**) (100 µg/kg), was studied in mice following i/v and i/p injections. The C_max_ of frondoside A in plasma after i/v injection was approximately seven-fold higher than after i/p injection, whereas the AUC after i/v injection was 4.8-fold higher than after i/p injection (Table 2). After i/v injection, the plasma concentration–time function was two-exponential with t_1/2 α_ of 2 min and t_1/2 β_ of 158 min. However, frondoside A was not stably detected in the plasma after peroral administration (100 and 500 μg/kg) in rats due to the sensitivity of the method (Table 1) [41].

A triterpene glycoside cucumarioside A_2_-2 (**12**) (cumaside), isolated from Far Eastern sea cucumber *Cucumaria japonica*, shows anticancer and immunostimulatory activities [100,102,103]. The pharmacokinetics of cumaside was studied after i/p and peroral administration in mice (5 mg/kg). Cumaside was quickly absorbed in blood after i/p administration (C_max_ = 114 μg/mL, T_max_ = 4 min). The absorption after peroral administration was rather slow (C_max_ = 75 μg/mL, T_max_ = 40 min) [104]. In contrast, Aminin reported a similar absorption of cumaside after i/p and peroral administration with relatively the same T_max,_ C_max_, and AUC (Table 2) [55]. 

#### 3.1.4. Sea Fungus-Derived Compounds

Marine fungi are a significant part of ocean biodiversity and have tremendous potential as a source of new drug candidates [105,106].

Sesterterpene 6-epi-ophiobolin G (**13**) (MHO7), produced by a mangrove fungus *Aspergillus ustus*, exhibited promising anticancer activity in 41 cell lines [107]. MHO7 was detected in plasma at 0.5 h after peroral administration in mice (500 mg/kg). The first peak concentration was achieved at 8 h (Table 2), followed by a gradual decrease up to 15 h. The second peak was observed at 10 h. This indicated a possible secondary absorption of sesquiterpene [45].

Diindolinonepyrane (**14**), 2,5-bis-[8-(4,8-dimethyl-nona-3,7-dienyl)-5,7-dihydroxy-8-methyl-3-keto-1,2,7,8-tertahydro-6H-pyran[a]isoindol-2-yl]-pentanoic acid (2,5-BHPA) isolated from marine fungi *Stachybotrys longispora*, possesses significant thrombolytic activity [108,109]. The linear pharmacokinetics was observed for 2,5-BHPA after i/v injection in rats at doses of 10–20 mg/kg. The concentration of 2,5-BHPA rapidly decreased in the blood after injection. The AUC was proportional to dose (412.2 and 899.1 μg/mL·min for 10 and 20 mg/kg, respectively) [37]. In another study, dogs were i/v-injected with 2,5-BHPA at doses of 2.5, 5.0, and 7.5 mg/kg. The AUC value increased linearly with increasing dose [40]. The shape of plasma concentration–time curves was the same as described previously for rats [37]. Low permeability and low recovery were observed for 2,5-BHPA in the Caco-2 model. The authors suggested that peroral administration is less appropriate than i/v injection [40].3.1.5. Marine Sponge-Derived Compounds.

Marine sponges (phylum *Porifera*) are the most basal type of multicellular marine organisms. About 8000 species of sponges have been described. They significantly contribute as unique, renewable, and rich suppliers of new and valuable drug candidates [110]. Sponges produce different classes of compounds that display anti-bacterial, anti-fungal, antiviral, anti-spasmodic, cytotoxic, and other bioactivities [23,111,112,113].

A sesquiterpene quinone ilimaquinone (**15**) (IQ), isolated from sponge *Hippiospongia metachromia,* exhibits anti-bacterial, antiviral, and anti-cancer activities [114,115,116]. Recently, the pharmacokinetics of IQ was reported in rats after peroral (10 mg/kg) administration. The C_max_ of 943 ng/mL was observed at 2.5 h post-administration [117]. Later, the same group of authors investigated the pharmacokinetics of the mixture of IQ and epi-IQ after i/v (3 mg/kg containing 2 mg/kg of IQ and 1 mg/kg of epi-IQ) and peroral (30 mg/kg containing 20 mg/kg of IQ and 10 mg/kg of epi-IQ) administration in rats. The AUC for IQ was approximately six-fold higher than that of epi-IQ after i/v injection. After peroral administration, C_max_ for IQ was 11.6-fold higher compared with epi-IQ, whereas the T_max_ for both substances was almost the same (Table 2) [46]. The absorption of ilimaquinone after the peroral administration of the mixture seems to be faster compared with single-compound administration [117]. However, this difference was not statistically significant. 

β-carboline alkaloid manzamine A (**16**), isolated from a sponge of *Haliclona* sp., exhibited potent antibacterial, anti-cancer, anti-malarial, and anti-HIV activities [56,118,119,120]. In a pharmacokinetics study, manzamine A isolated from a sponge of the *Acanthostrongylophora* genus was administered to rats by i/v (10 mg/kg) and peroral (50 mg/kg) routes. After i/v injection, the plasma concentration of manzamine A was about 40 μg/mL. The plasma concentration–time function was exponential. After peroral administration, manzamine gradually increased in plasma and reached C_max_ of 1066 ng/mL at a T_max_ of 10 h. Manzamine A has good solubility in acids. This is helpful for its absorption in the stomach. The logP (8.34) determined during chromatographic method development suggests a reasonable permeability for this alkaloid [56].

#### 3.1.5. Sea Urchin-Derived Compounds

Sea urchins are marine invertebrates belonging to *Echinoidea*. All parts of these organisms have been used in traditional medicine [121] and are a highly valuable source of medicines. The sodium salt of naphthoquinone pigment echinochrome A (**17**) (under the name of histochrome), isolated from the shell of sea urchin *Scaphechinus mirabilis*, is approved in Russia for the treatment of acute myocardial infarction and, in ophthalmology, for dystrophic diseases of the retina and cornea [9,122,123]. The absorption of histochrome was studied in rabbits after subconjunctival and parabulbar injection. It was not absorbed into the blood, but rapidly distributed over the tissues of the eye [124]. 

A glycopeptide (GPP) from the internal organs of the green sea urchin *Strongylocentrotus droebachiensis* is effective as an anti-inflammatory agent in the treatment of bronchitis [125]. GPP was administered to rats i/v (100 µg/kg) and intranasally (i/n) at the dose of 50–200 µg/kg. Its plasma concentration–time function was two-exponential after i/v administration with a mean T_1/2_ of 0.8 h and an AUC of 8.00 µg·h/mL. After i/n administration, GPP appears in plasma at 15 min and reaches a C_max_ of 2.90–6.22 µg/mL at a T_max_ of 0.67–0.75 h with AUC of 7.14 µg·h/mL. The C_max_ increases with increasing dose; however, it does not change after repeated dose administration. Repeated doses of i/n administration (3 days of 100 μg/mL) led to a significant increase in AUC (up to 23 µg·h/mL. Notably, C_max_ and AUC were equal after both administration routes at identical doses (Table 2). This finding supports the rationality of the i/n administration of GPP [30].

Lipid extract of the gonads from *S. droebachiensis* (LES) lowered glucose level and inhibited enzyme dypeptydylpeptidase IV (DPPIV) [126]. The inhibition of DPPIV was used as a biomarker for the pharmacokinetics of LES in rabbits after peroral administration (Table 1). The pharmacokinetic was linear at doses of 5–25 mg/kg. The C_max_ of LES in plasma was 37.1–114.7 µg/mL at T_max_ of 3–3.5 h, but the difference in T_max_ with dose increase was not statistically significant. A second increase in LES concentration in plasma was observed at 10 h. The AUC ranged from 192.3 to 594.2 µg·h/mL with increasing dose (Table 2) [29].

#### 3.1.6. Marine Compounds Derived from Other Species

Aplidine (**18**) (syn. dehydrodidemnin B, plitidepsin) is a cyclodepsipeptide with potent anticancer activity isolated from Mediterranean tunicate *Aplidium albicans* [127]. The absorption of aplidine was studied in rats. Two minutes after i/v injection at the dose of 700 μg/kg, the concentration of aplidine in blood was 100 ng/mL [44].

Kahalalide F (**19**) is a cyclic depsipeptide found in Hawaiian mollusk *Elysia rufescens* and the green algae *Bryopsis* sp. [128]. This compound has considerable potential as an anticancer drug [129,130]. The pharmacokinetic of kahalalide F was studied in mice. After an i/v injection of 278 μg/kg, the initial plasma concentration of kahalalide F was 1.55 μM. The plasma concentration–time function was two-exponential with t_1/2 α_ of 15.8 min and t_1/2 β_ of 4.4 h [57].

Dolastatins 10 (**20**) and 15 (**21**) are small peptides, isolated from the shell-less mollusks sea hare *Dolabella auricularia*, that have shown anticancer activities [131]. The pharmacokinetics of radio-labeled dolastatin 10 was studied in mice after i/v (0.24 mg/kg), s/c (0.32 mg/kg), and i/p administrations. A rapid decrease in dolastatin in plasma was observed after i/v injection. The dolastatin concentration in plasma after s/c injection increased slowly up to a C_max_ of 11 ng/mL. The human, dog, and mouse plasma protein-binding for dolastatin was calculated as more than 81% [58]. After i/v injection in mice (1 mg/kg), both dolastatins (10 and 15) were detected in plasma at 5 min post-injection. The plasma concentration–time function was two-exponential with t_1/2 α_ of 0.04 h and 0.09 h and t_1/2 β_ of 1.6 h and 0.52 h for dolostatins 10 and 15, respectively. The AUC dolastatin 10 was 1.6-fold higher than for dolastatin 15. The plasma binding of dolastatin 10 was more than 90% [59]. 

Bryostatin 1 (**22**) is a cyclic macrolide isolated from a marine bryozoan, *Bugula neritina* [132]. It is known as an anticancer compound, a modulator of protein kinase C, and is used as a neurodegenerative agent [133,134]. In a pharmacokinetics study, bryostatin 1 was i/v- and i/p-injected in mice at the dose of 40 μg/kg. Following i/v injection, bryostatin 1 appeared in plasma at C_max_ of 92.9 ng/mL. The plasma concentration–time function was two-exponential with t_1/2 α_ of 1.05 h and t_1/2 β_ of 1.6 h and 22.97 h. After i/p injection, C_max_ was 13.5 ng/mL. Although the C_max_ after i/p injection was lower than after i/v injection, the AUC after i/p injection was 1.6-fold higher than after i/v injection (Table 2). These data provide evidence of good absorption and retention in blood after i/p injection [63]. In another study, the pharmacokinetics of bryostatin 1 was evaluated after i/v injection in mice at doses of 3.50 and 5.25 μg/kg. The peak concentration in plasma increased with increasing dose. The C_max_ value was about 2.26 ng/mL at a high dose [135].

### 3.2. Distribution

#### 3.2.1. Algal-Derived Compounds

The distribution of fucoidan (**1**) was reported after i/v, peroral, and transdermal applications. Equal values of MRT of 5.48 ± 1.78 h and 5.30 ± 1.65 h were observed for fucoidan with an MW of 100 kDa after i/v injection and peroral administration, respectively [35]. The high-MW fucoidan, after peroral administration to rats, showed prolonged MRT in blood (6.79 h) and accumulation in organs with a filtering function, such as spleen, kidneys, and liver (Table 3) with MRT of 14.57, 12.39, and 9.26 h, respectively [27]. Similar results were observed after i/v injection of FITC-labeled commercially available fucoidan in mice. Fucoidan quickly distributed to the liver and kidney and reached the maximal concentration at a T_max_ of 0.5 h. The MRT values in blood, liver, spleen, lung, and kidney were 3.23, 14.66, 303.96, 6.88, and 20.14 h, respectively [32]. The maximal accumulation was observed in the kidney. However, it was not detected in the brain or the heart (Table 3) [32]. These data support the renoprotective effects of fucoidan that were reported previously [61,136]. After topical application in rats, a high concentration of fucoidan was observed in skin in 15 min, which started to decrease in 1 h. This indicates that, initially, the epidermis was saturated with the drug. However, after one hour, the concentration of fucoidan increased in muscles and plasma with muscle–plasma concentration ratios of 1.77 and 1.75 at the first and second hours postdose, respectively. The linear pharmacokinetics was observed at the doses of 50–150 mg/kg [28].

The mean V_ss_ for GRFT after i/v injection was 0.5 L, without statistically significant dose dependence. After s/c injection, V_ss_ decreased from 1.2 to 0.2 L at 10 and 20 mg/kg, respectively [51]. The maximal amount of GRFT was observed in the spleen, followed by the kidney and liver of mice after multiple-dose s/c injection. Due to intensive accumulation of GRFT in spleen, the authors advised to control the immune response elicited by GRFT treatment to prevent GRFT immune-related toxicity [68].

After i/p and s/c injection in mice, sodium alginates were found in the spleen, liver, and kidneys [73].

After i/v injection (139 mg/kg) in mice, halomon (**3**) was quickly absorbed and distributed to tissues. At 5 min after injection, the maximal concentration of halomon was detected in the heart, followed by lungs, kidney, brain, liver, spleen, skeletal muscles, and fat. Notably, halomon crossed the blood–brain barrier. Its concentration in the brain was comparable with concomitant concentrations in plasma and other tissues. The lipophilic nature of halomon and multiple halogens in the structure could explain its remarkable persistence in fat tissue. This quick and wide distribution may explain the rapid reactivity of halomon to cancer cells in vitro [33]. 

The tissue distribution of fucoxanthin (**6**) and its metabolites (fucoxanthinol (**23**) and amarouciaxanthin A (**24**)) was studied in mice after peroral administration of fucoxanthin. The maximal concentration of both metabolites was detected in the liver, followed by lung, kidney, heart, and spleen. Interestingly, this order in tissue distribution was the same for both metabolites. Fucoxanthin was also detected in all the above-mentioned tissues. High concentrations were found in the heart and liver. In adipose tissue, amarouciaxanthin A was more abundant. Fucoxanthinol and amarouciaxanthin A were present in adipose tissue until 72 h after the administration of fucoxanthin. However, in other tissues, these metabolites were not detectable after 24 h [80]. The mean distribution volume of fucoxanthin after i/v injection in rats was much lower than the V_ss_ of its metabolite fucoxanthinol (0.7 L/kg vs. 8.8 L/kg). After peroral administration, the difference in V_ss_ for compounds was not statistically significant [42]. Taken together, liver, heart, and adipose tissues have been identified as targets for the biological activities of fucoxanthin and its metabolites. These findings support the anti-obesity, neuro- and cardio-protective, and hepatoprotective effects of fucoxanthin [137,138,139].

The tissue distribution of astaxanthin (**6**) was studied in rats fed a diet with 3% astaxanthin ad libitum. The maximal accumulation of astaxanthin was detected in the spleen, followed by kidney and adrenals after 14 days of the experiment. The concentrations in the liver, heart, lung, and eyes increased but were not as high as in other organs [140]. In another study [52], MRT and the V_ss_ of astaxanthin in plasma after i/v injection in rats were not statistically significantly changed with increasing dose (5–20 mg/kg). Astaxanthin was widely distributed to all organs listed in Table 3 at 8 and 24 h after peroral administration (100 mg/kg) in rats. However, the distribution in organs, particularly the spleen and liver, was more uniform than in previous study [140]. The broad tissue distribution evidences the high affinity of organs for astaxanthin and supports its multifunctional pharmacological activity [52].

#### 3.2.2. Crustacean-Derived Compounds

The commercial ^125^I-labelled chitosan (**7**) (with MW = 5–10 kDa) was found in the liver, lungs, spleen, kidney, hearth, urine, and thyroid 5 min after i/v injection in rats. At one hour after injection, it was still detected in all organs, with predominant accumulation in the liver (particularly high-MW chitosan) and urine [93]. In another study, 50% deacetylated fluorescein isothiocyanate (FITC)-labeled commercial chitosan was i/p injected in mice at the dose of 29 mg/kg. Chitosan was quickly absorbed and distributed. At one hour after injection, it was practically not detected in blood, and the maximal concentration was in the kidney, followed by liver, abdominal dropsy, and spleen. At 24 h post-injection, chitosan was detected in significant amounts in the kidney, spleen, and liver [141]. The FITC-labeled commercial chitosan with an MW of 3.8–230 kDa was perorally administered to rats. Chitosan with an MW of 3.8 kDa was detected in the epithelium of villi in the duodenum and jejunum compartments. Chitosans with an MW 3.8 and 7.5 kDa were found in the submucosa levels as well. Chitosan with a 230 kDa MW was not detected in the gastrointestinal tract [53]. The FITC-labeled chitosan (MW = 990 Da–76 kDa) was found in the heart, kidney, liver, lungs, spleen, and thymus after peroral administration in mice. The maximal concertation of chitosan was observed in the liver and kidney, indicating its accumulation and future elimination pathways. Low- and high-MW chitosan increased in the liver during the first 30 min, followed by a decrease to 4 h after administration, whereas the concentration of chitosan with an MW of 33–39 kDa reached a maximum in the liver at 2 h and was constant up to 4 h post-administration [54].

#### 3.2.3. Sea Cucumber-Derived Compounds

Both oligoglycosides EA (**8**) and HA (**9**) were found in the liver after peroral administration in rats. The concentration–time functions of EA in the liver are characterized by two maxima with C_max_ of 118.6 ng/g at T_max_ of 2 h and C_max_ of 121.2 ng/g at T_max_ of 9 h. Only one peak (C_max_ = 70.9 ng/g, T_max_ = 9 h) was observed for HA. The concentration of both compounds decreased at 11 h. Notably, approximately 50% growth in the concentration of both compounds was observed at 24 h [39]. A subsequent study by the same authors identified that both oligoglycosides and their metabolites can be absorbed by the rat intestine [142].

The volume of distribution of frondoside A (**11**) after i/p injection in mice was more extensive than after i/v injection (28 vs. 0.87 L/m^2^) [41].

Cumaside (**12**) was quickly distributed to the organs. It was detected in the stomach, heart, kidney, liver, and spleen 4 min after i/p administration in mice and 10 min after peroral administration (Table 3) [104]. The maximal V_ss_ was found in the blood at 1706 µL and 2460 µL after i/p and peroral administration, respectively. The largest AUC was in the heart (10490 µg·h/mg, MRT = 123 h) after i/p injection and in the stomach (7560 µg·h/mg, MRT = 1.75 h) after peroral administration [55]. Considering the immunostimulatory activity of cumaside, Pislyagin et al. [47] studied the pharmacokinetic parameters of this compound in the spleen after i/p administration in mice at the dose of 5 mg/kg. Cumaside was detected in the spleen 5 min after administration, followed by a fast increase up to a C_max_ of 91.72 ng/mg (T_max_ = 10 min). The AUC was 104.38 ng·min and MRT was 134.5 min. 

#### 3.2.4. Sea Fungus-Derived Compounds

The MRT of sesterterpene MHO7 (**13**) in plasma after peroral administration in mice was 8.76 h. The concentration of MHO7 in the stomach was about three-fold higher than in the intestine, while the T_max_ in both organs was the same (8 h). The quantities of MHO7 in the stomach and intestine were 16.8% and 5% of the administered dose, respectively. Additionally, MHO7 was detected in the reproductive organs, fat, kidney, liver, lung, muscles, brain, heart, and spleen (Table 3). Notably, the highest concentration of MHO7 within 8–30 h after administration was observed in the reproductive organs. These data identify reproductive organs as a target for the sustained effect of the HMO7 formulation [45].

The MRT of 2,5-BHPA (**14**) in the plasma of rats after i/v injection was very low (10 min) and equal for both doses (10 and 20 mg/kg). 2,5-BHPA was widely distributed after i/v injection in rats. Its maximal concentration was observed in the liver, followed by the kidney, spleen, intestine, lung, stomach, and heart (Table 3). The compound was not detected in the brain [37]. After i/v injection in dogs (2.5–7.5 mg/kg) [40], the MRT was approximately 2.5-fold higher compared with the MRT in rats [37]. However, it was not dose dependent. After i/v injection in dogs, the concentration of 2,5-BHPA in liver and bile was approximately seven-fold higher than in kidney, lung, stomach, spleen, muscle, heart, and intestine (Table 3). In contrast with previous data on rats [37], 2,5-BHPA crosses the blood–brain barrier in dogs and was found in significant amounts in the brain. The concentrations in urine and feces were similar [40].

#### 3.2.5. Marine Sponge-Derived Compounds

Manzamine A (**16**) was characterized by a large apparent volume of distribution (V_ss_ = 23.7 L/kg) after i/v injection in rats at a dose of 10 mg/kg [56].

#### 3.2.6. Sea Urchin-Derived Compounds

The distribution period of echinochrome A (**17**) was shorter than the elimination period. Echinochrome A was detected in the cornea, crystal of the eye, vitreous humor, retina, and intraocular fluid after both subconjunctival and parabulbar injections in rabbits. The highest concentration of echinochrome A was found in the choroid with AUCs of 256.26 and 193 μg·h/mL after subconjunctival and parabulbar injections, respectively. The T_max_ varied between 0.35 and 1.6 h after subconjunctival injection and 0.57 and 1.3 h after parabulbar injection. Subcutaneous injection resulted in a high accumulation of the drug in the choroid, cornea, and retina with MRTs of 2.5, 2.43, and 2.08 h, respectively. After parabulbar injections, the maximal accumulation of the drug was observed in the crystal of the eye, retina, and vitreous humor with MRTs of 2.15, 1.96, and 1.64 h, respectively [124]. Later, Guseva et al. confirmed that echinochrome A can cross the blood–ocular barrier in rabbits after intravenous, subconjunctival, and parabulbar injections, and was detected in the vitreous humor and intraocular fluid [143].

The MRT of GPP in plasma after i/v injection in rats was 1.11 h. A more prolonged MRT of 5.58 h was observed after i/n administration. Repeated doses of i/n administration led to an increase in MRT of approximately 10-fold. After i/n instillation, the maximal concentration of GPP was found in the nose mucosa (C_max_ = 53.66 µg/g, AUC = 248.75 µg·h/g, MRT = 8 h), followed by the spleen (C_max_ = 2.53 µg/g, AUC = 28.90 µg·h/g, MRT = 10.2 h) and adrenal glands (C_max_ = 2.67 µg/g, AUC = 27.06 µg·h/g, MRT = 21.42 h). The concentrations in striated muscle, kidneys, and liver were significantly lower (Table 3) [30].

The MRT of LES in plasma after peroral administration in rabbits (5–25 mg/kg) was relatively prolonged (10.7–14.3 h). However, the difference was not statistically significant with increasing dose [29].

#### 3.2.7. Marine Compounds Derived from Other Species

About 50% of aplidine (**18**) after i/v injection in rats was stored in blood cells [44].

The V_ss_ for kahalalide F (**19**) after i/v injection was more than 100 times the body weight, suggesting that the drug is widely distributed in peripheral tissues [56]. 

The V_ss_ for bryostatin 1 (**22**) was equal after i/v and i/p injection, while the MRT after i/p injection was almost twice that compared with i/v injection. After both routes of administration, bryostatin 1 was detected in the kidneys, brain, fat, thymus, liver, lung, spleen, bone marrow, lymph nodes, skeletal muscles, and the gastrointestinal tract. The highest concentration of the drug was observed in the liver, lung, and bone marrow (Table 3) [59]. 

Another study showed that the brain concentrations of bryostatin 1 after i/v administration in mice at the doses of 3.50 and 5.25 μg/kg were not significantly different. This indicated that the dose of 3.5 μg/kg saturates the brain. At 4 h post-injection, the brain concentrations at 3.50 and 5.25 μg/kg were 42% and 30% of the plasma, respectively. The peak of brain protein kinase C activation was observed at 0.5 h [134]. Bryostatin 1 apparently crosses the blood–brain barrier, and the abovementioned results support the use of bryostatin 1 in the treatment of neurodegenerative diseases.

### 3.3. Metabolism

#### 3.3.1. Algal-Derived Compounds

For a better understanding of the possible sites for the metabolism of fucoidan (**1**), rats were fed a diet of fucoidan supplemented with *N*-butyl-*N*-(4-hydroxybutyl) nitrosamine (NN4HN). NN4HN intensifies the intestinal absorption of fucoidan [144]. The immunohistochemical assay revealed the presence of fucoidan in the small intestine, the epithelial cells of the jejunum, and the mononuclear cells of the lamella gland of the jejunum [145]. Suda et al. reported the involvement of Kupffer cells (specialized liver macrophages) in the metabolic degradation of β-D-glucans [146]. As a ligand for macrophage scavenger receptor-A [147], fucoidan was accumulated by Kupffer cells [145].

GRFT was found in unchanged forms in the feces and urine of rats after i/v, s/c, and peroral administrations [51].

Some sugars, such as D-glucose, are resorbed by renal tubules after filtration by the kidney [148]. As soon as alginate oligosaccharide (**2**) was not detected in the plasma 2 h after peroral administration, the authors suggested that it is not resorbed by the renal tubules [43].

The metabolism of halomon (**3**) was studied in vitro in mice and human liver cells. The authors found that halomon is metabolized by mouse and human hepatic cytochrome P-450 enzymes, resulting in the loss of one chlorine atom and one bromine atom [75]. These data suggest the hepatic metabolism of the drug, consistent with the pharmacokinetic data previously obtained by the same group of authors [33].

As a result of hydrolysis by digestive enzymes, lipase and cholesterol esterase, in the gastrointestinal tract, fucoxanthin (**5**) converts to fucoxanthinol (**23**) and is absorbed by the intestine [149]. Then, it is metabolized to amarouciaxanthin A in the liver by short-chain dehydrogenase/reductase [150]. Fucoxanthin is converted into fucoxanthinol by deacetylation [149,151]. Fucoxanthin is quickly metabolized into fucoxanthinol after both i/v and peroral administration in rats as evidenced by LC-MS/MS analysis [42]. This evidences the important contribution of fucoxanthinol to the pharmacological activity of fucoxanthin. 

#### 3.3.2. Crustacean-Derived Compounds

The experiments on mice i/p injected with FITC-labeled chitosan (**7**) showed that it should easily undergo enzymatical degradation in body fluids or tissues because at one hour after injection, it was practically not detected in the blood. However, it accumulated in the kidney and the urinary tract, where it may undergo further enzymatic degradation [141]. Water-soluble chitosan could be digested by the enzymes in the upper intestine. However, these dissolved macromolecules would gradually precipitate with increasing pH in the lower intestine. This prevents further degradation of chitosan by the enzymes, so chitosan is not absorbed by the intestine [54].

#### 3.3.3. Sea Cucumber-Derived Compounds

The metabolism of oligoglycosides EA (**8**) and HA (**9**) was studied in an in vitro model by incubation with intestinal microflora and in an in vivo study on rats after peroral administration. In vitro, six deglycosylated metabolites of oligoglycosides (M1–M6) were identified, with the prevalence of M6 for EA and M2 for HA. In vivo, metabolites were identified in serum (4), urine (6), and feces (4). The authors concluded that glycosylation is the main intestinal microflora-mediated metabolic pathway for oligoglycosides [142]. 

After i/p injection, cumaside was stable, and none of its metabolites were observed in spleen homogenate or the intact organs within 24 h [47].

#### 3.3.4. Sea Fungus-Derived Compounds

The concentration of sesterterpene MHO7 (**13**) in the liver 1 h after oral administration in mice was apparently higher than in other organs. This suggests that MHO7 metabolites first occur in the liver [45].

#### 3.3.5. Marine Sponge-Derived Compounds

The metabolites of manzamine A (**16**) were not detected in rats after i/v and peroral administration. Experiments in human liver microsomes suggest the possibility of slow hepatic metabolism of manzamine A with a predicted low hepatic extraction ratio of 0.4 [56]. 

#### 3.3.6. Sea Urchin-Derived Compounds

The metabolism of 7-ethyl-2,3,5,6,8-pentahydroxy-1,4-naphthoquinone (echinochrome A) (**17**) was studied in rats after subcutaneous injection (10 mg/kg, 10 consecutive days). Echinochrome A was established as completely metabolized in rats to 3-methoxy-2,5,6,8-tetrahydroxy-7-ethyl-1,4-naphthoquinone and 2-metoxy-3,5,6,8-tetrahydroxy-7-ethyl-1,4-naphthoquinone [152].

#### 3.3.7. Marine Compounds Derived from Other Species 

Aplidine (**18**) was detected in urine in an unmodified form after i/v injection in rats [44].

After injection of dolastatin 10 (**20**) in mice, a significant part of the radioactivity in plasma was attributed to radiolabeled metabolites. It was established in vitro that dolastatin 10 is rapidly metabolized to the more polar dihydroxy derivative after incubation with a homogenate of whole rat liver [58]. Later, *N*-dimethyl dolastatin 10 was detected in the plasma of patients during phase I of clinical trials [153].

After i/v injection of bryostatin 1 (**22**) in mice, one major and a few minor metabolites were detected in plasma by HPLC. However, they were not identified. The same major metabolite was detected in kidneys and lungs. The excretion of bryostatin 1 in feces within 72 h provides insight that it may undergo enterohepatic circulation [60].

### 3.4. Elimination

#### 3.4.1. Algal-Derived Compounds

The Cl of fucoidan (**1**) with an MW of 100 kDa was 0.0037 mg/μg/mL/h after i/v injection and 0.138 mg/μg/mL/h after peroral administration in rats [35]. The Cl of FITC-labeled commercially available fucoidan was 0.25 mg/(μg/g)/h after i/v injection in mice [32]. Low-MW fucoidan was not detected in serum 6 h after i/v injection in rabbits [36]. The concentrations of low- and medium-MW fucoidans from *L. japonica* in urine 48 h after peroral administration in rats (800 mg/kg) were 1206.0 and 874.4 μg, respectively [34]. The authors suggested that due to the rapid penetration and retention in the skin, fucoidan showed prolonged T_1/2_ (20.75 ± 9.43 h) in plasma and low bioavailability (F = 17.7% ± 7.7%) after topical application in rats [28]. Fucoidan with an MW of 100 kDa was characterized by equal T_1/2_ (4.01 and 4.1 h, respectively) after i/v injection and peroral administration in rats. The bioavailability was 8.91% [35]. The FITC-labeled commercially available fucoidan was not detected in blood 4 h after i/v injection in mice [32].

The difference in T_1/2_ after i/v and s/c injections of GRFT at 10 mg/kg was statistically not significant, whereas an increase in dose up to 20 mg/kg led to a delay in elimination by approximately 2.6-fold after s/c injection when compared with i/v injection. GRFT was still detectable in the serum of rats after both i/v and s/c injection (10 mg/kg) up to 96 h. The bioavailability of GRFT after s/c injection was dose dependent (F = 43% at 10 mg/kg and 90% at 20 mg/kg). GRFT was found in feces and urine of rats within 24 h following i/v and s/c injections. The concentration of GRFT in urine after peroral administration was below the detection limit, but it was found in feces. GRFT was not orally bioavailable even after multiple doses of treatment. However, after multiple doses of peroral administration, GRFT was found in feces and urine [51]. About 51 ng/mL of GRFT was found in the plasma of mice after s/c injection (50 mg/kg) at 24 h posttreatment. GRFT was still detectable through day 14 after treatment [68].

After i/p injection of sodium alginate in mice, the T_1/2_ was 12.5 h. The bioavailabilities of sodium alginate were 44% and 5% following i/p and s/c injections, respectively. Alginates were excreted with urine [73]. Alginate oligosaccharide (AOs) after peroral administration in mice was eliminated with urine with a C_max_ of 425 µg/mL at 30 min, followed by a rapid decrease. After 6 h, AOs was still detectible in urine [43]. In another study, eight fractions of sodium alginate oligosaccharide were obtained by lyase enzyme degradation. The P8 fraction was administered to rats at 3 g/kg. At 24 h after administration, about 60% of P8 was excreted in the feces and 5% in the urine [154]. About 73–79% of alginic acid and its salts were excreted by rats in feces 72 h following peroral administration (90.4 mg/kg) [155]. About 86–91% of sodium alginate labeled with 14C was excreted in rat feces after 17 h of feeding a diet containing about 10% alginate. About 0.1–0.2% of alginate was eliminated with urine, whereas only 0.002–0.007% was found in plasma [156]. 

Halomon (**3**) was eliminated with a Cl of 36–56 mL/min kg after i/v injection. The clearance was non-linearly dose dependent at doses of 20–135 mg/kg. Halomon bioavailability was 45%, 47%, and 4% after i/p, s/c, and peroral administration, respectively [33]. The urinary excretion of halomon was minimal (0.0034–0.0046%). Its hepatic concentration decreased rapidly and was relatively low compared with other tissues and plasma. Therefore, the authors suggested biliary excretion or hepatic metabolism as the major routes of halomon elimination [33].

The elimination of fucoxanthinol (**23**) (T_1/2_ = 4.5 h) was faster than amarouciaxanthin A (**24**) (T_1/2_ = 6.7 h) after peroral administration of fucoxanthin (**6**) in mice. Both metabolites were relatively quickly eliminated from the liver (T_1/2_ = 2.5 h). Its kidney concentration decreased much slower (T_1/2_ = 6.3 h for fucoxanthinol and 10.1 h for amarouciaxanthin A). The most prolonged terminal half-life time was found for adipose tissue (T_1/2_ = 16–25.5 h) [80]. The difference in T_1/2_ for fucoxanthin after i/v and peroral administration in rats was statistically not significant (Table 2), whereas the Cl after peroral administration was much quicker. The clearance of fucoxanthin after peroral administration was about 30-fold faster comparing with its metabolite [42].

The Cl of astaxanthin (**6**) after i/v injection to rats at 20 mg/kg was by 66% slower than at 5 mg/kg. Astaxanthin was eliminated predominantly via non-renal clearance [52]. After peroral administration in rats, the concentration of astaxanthin in the kidney was much higher than in the liver. The authors suggested that the kidneys play the principal role in the excretion of the substance, even though the liver is the key site of metabolism [52,140].

#### 3.4.2. Crustacean-Derived Compounds

The experiments on mice i/p injected with FITC-labeled chitosan (**7**) showed that it is quickly eliminated in urine [141]. The rapid plasma clearance of commercial ^125^I-labelled chitosan was observed after i/v injection in rats. Its concentration decreased by 1.5- and 3-fold (for low- and high-MW chitosan, respectively) at 1 h. At the same time, the concentration in urine rapidly increased. The most intensive increase was found for low-MW chitosan. This indicates urine as being the most likely elimination pathway for chitosan [93]. All observed data about the pharmacokinetics of chitosan suggest that it has no significant accumulation in the body after peroral or other routes of administration. Together with the non-toxicity of chitosan, these data suggest its potential for use as a transporter for improving the penetration of pharmaceutical formulations.

#### 3.4.3. Sea Cucumber-Derived Compounds

The elimination of EA (**8**) and HA (**9**) isolated from *Pearsonothuria graeffei* was very rapid after i/v injection in rats. The concentration of EA in plasma decreased approximately 10.5-fold in 15 min and remained stable (0.2 µg/mL) two hours after injection. The elimination of HA was slower than EA. Its concentration decreased 10.5-fold in approximately one hour after injection and remained steady until two hours post-administration. The authors suggested that the difference in clearance was due to the specificity of oligoglycosides’ structures and the higher concentration of HA in the extract, whereas the final concentration of both compounds was nearly similar. The detection of EA and HA in the serum of rats within 24 h indicated its slow elimination after peroral administration [39]. The concentrations of both oligoglycosides EA and HA in the intestine of rats reached a maximum at 2 h after peroral administration of extract (300 mg/kg). Then, their concentrations decreased, followed by the second maximum at six hours. The authors associated this finding with the enterohepatic circulation and secretion of oligoglycosides with bile acid into the intestine. The concentration of metabolites decreased in the intestine 6 (metabolites of HA) and 4 h (metabolites of EA) after extract administration. Both oligoglycosides and all their six metabolites were found in urine. Notable, the concentration of EA was significantly higher than that of HA. Conversely, the metabolites of HA were more abundant than those of EA. This suggests that EA is more easily excreted in urine. The authors explained the absence of metabolites with the one sugar moiety in the feces by its relatively high absorption rate in the gut [142]. Both saponins EA (**8**) and HA1 (**9**) were detected in plasma 24 h after i/v injection in rats, whereas the T_1/2_ of HA1 was half that T_1/2_ of EA. The T_1/2_ of EA after peroral administration was relatively shorter (~23%) compared with i/v injection (Table 2). The bioavailability of EA was 59%. HA1 was not detected in the plasma after oral administration [38].

The T_1/2_ of frondoside A (**11**) after i/p injection was 1.6-fold longer compared with that after i/v injection. The body Cl was 6.35 mL/min/m^2^ after i/v injection vs. 127 mL/min/m^2^ after i/p injection. The calculated F was 20% after i/p administration of frondoside A [41].

After peroral administration in mice, cumaside (**12**) was rapidly eliminated with the T_1/2_ ranging from 0.3–1.4 h, and CL varied within 793–3519 µg/min depending on the organ. The i/p administration was followed by significantly prolonged T_1/2_ (9–22 h) and delayed Cl (18.7–94.3 µg/min) depending on the organ [56,105]. Cumaside was slowly eliminated from the spleen of mice after i/p administration with a Cl of 21.5 mL/min and T_1/2_ of 91.6 min [47]. During pharmacokinetic studies, the absorption rate of cumaside and its distribution were the same after i/p and peroral administration in mice. It does not accumulate and is quickly eliminated from the organs after peroral administration. Based on these data, peroral administration was suggested as a rational method for future clinical trials [55,104].

#### 3.4.4. Sea Fungus-Derived Compounds

The sesterterpene MHO7 (**13**) circulated for a relatively long time in the blood (T_1/2_ = 7 h and CL = 47.61 L/h/kg) after peroral administration in mice. The high concentrations of MHO7 in the kidney 12 h after administration suggested renal excretion as the dominant elimination route [45].

2,5-BHPA (**14**) was rapidly eliminated from rat plasma after i/v injection. The clearance (about 0.023 (mg/kg)/(μg/mL)/min) and T_1/2_ (about 22 min) were equal for both doses studied. At 15 min after injection, 2,5-BHPA reached a maximal concentration in the liver. At 60 min after injection, 2,5-BHPA was not detected in the plasma or other organs except for the liver and small intestine. This may indicate enterohepatic circulation [37]. The elimination of 2,5-BHPA from the plasma of dogs was about twice as long compared with rats (Table 2), and clearance was delayed three-fold when compared with the experiment on rats. The significant amount of 2,5-BHPA in urine and feces one hour post-injection may indicate excretion routes, but this aspect requires additional experiments [40].

#### 3.4.5. Marine Sponge-Derived Compounds

The elimination of ilimaquinone (**15**) (IQ) after peroral (10 mg/kg) administration to rats was relatively quick (T_1/2_ = 1.2 h, Cl = 316 mL/h/kg). However, IQ was still detectable 24 h post-administration [115]. The elimination of IQ and epi-IQ after i/v injection of their mixture was much faster (T_1/2_ = 0.4–0.6 h) compared with peroral administration. The Cl for epi-IQ was three-fold greater compared with IQ (Table 2). Two hours after i/v injection, the concentrations of both IQ iso-forms were below the detection limit (Table 1). The elimination half-life time for IQ after peroral administration of the mixture was prolonged by approximately three-fold compared with the peroral administration of IQ alone. Although the T_1/2_ values for both iso-forms were equal after mixture administration, the clearance of epi-IQ was much greater (Table 2). The absolute bioavailability after peroral administration of the mixture was calculated as 38% and 13% for IQ and epi-IQ, respectively [46]. The results of this study highlight the importance of pharmacokinetic investigations of stereoisomers. This study indicated that ilimaquinone shows higher absorption and faster elimination compared with its epi-form. This specificity must be considered in the formulation development phase.

The alkaloid manzamine A (**16**) circulated in the blood for a long time after i/v injection, with T_1/2_ of 53.7 h and a slow Cl of 5.1 mL/min/kg. About 100 ng/mL of manzamine A was detected in plasma 96 h after both administration routes. The absolute bioavailability after peroral administration to rats was calculated to be 20.6%. This reasonable bioavailability could be explained by the metabolic stability and good absorption of manzamine A [56]. The results of pharmacokinetics investigations (long T_1/2_ and low plasma clearance) are important for pharmacodynamic implications considering the previously reported promising antimalarial activity of this compound [119].

#### 3.4.6. Sea Urchin-Derived Compounds

The T_1/2_ for echinochrome A (17) in eye tissues ranged between 0.53 and 1.73 h after subconjunctival injections and between 0.86 and 1.47 h after parabulbar injection in rabbits [124]. Echinochrome A was excreted by kidneys after subcutaneous injection in rats [152]. 

The elimination of GPP from rat plasma after i/v and i/n administration was relatively quick (T_1/2_ of about 0.8 h). Repeated doses of i/n administration (3 days of 100 μg/mL) led to approximately three times longer in T_1/2_. Because of the wide variably in the data, the difference between the T_1/2_ of GPP in plasma after i/v, and in plasma, liver, kidneys, striated muscle, and nose mucosa after i/n administration, was not considered statistically significant. GPP was not detected in the liver and kidneys 8 h after administration. In contrast, T_1/2_ was most prolonged in adrenal glands at up to 14.7 h [30].

About 8–11% of LES was still detected in the plasma of rabbits 24 h after peroral administration. A slight decrease in mean T_1/2_ (from 9.9 to 7.9 h) was observed for LES with dose escalation from 5 to 25 mg/kg, although it was statistically not significant [29].

#### 3.4.7. Marine Compounds Derived from Other Species 

Aplidine (**18**) quickly disappeared from blood after i/v injection with a T_1/2_ of 9 min. It was detectable at 20 min, but the amount was lower than the limit of quantification (5 ng/mL). Its concentration in urine was 0.3% of the injected dose after 24 h [44].

Kahalalide F (**19**) was not found in the plasma of mice 24 h after i/v injection and did not accumulate after repeated injections at intervals of 24 h. The total body Cl of kahalalide F was 23% of the blood flow in the liver. This evidenced that kahalalide F is rapidly eliminated from plasma and has low binding to extravascular tissues [57].

After i/v injection in mice, dolastatin 10 (**20**) was eliminated from plasma with a T_1/2_ of 5.6 h. After s/c injection, the elimination was faster (T_1/2_ = 3.7 h). Less than 2% of injected dolastatin 10 was excreted with urine in its unaltered form irrespective of the route of administration [58]. Both dolastatins (10 and 15) were rapidly eliminated from the plasma of mice after i/v injection. The elimination speed of dolastatin 15 was significantly shorter compared with that of dolastatin 10. Dolastatin 10 was still detected in the plasma of all animals at seven hours post-injection, and in the plasma of two of three animals at 24 h [59]. 

The value of plasma clearance of bryostatin 1 (**22**) after i/v injection in mice was about double the clearance after i/p injection. During the first six hours after i/v injection, about 19% bryostatin 1 was eliminated with urine. After 12 h, about 23% of the injected dose was eliminated with urine. The excretion with feces became important within 72 h after injection. At this time point, the elimination volumes of bryostatin 1 with urine and feces were equal. The excretion with feces within 82 h was about 80% of the injected dose. After i/p injection, about 13% of the drug was eliminated with urine at 12 h, and about 35% was eliminated within 72 h. The excretion with feces was 18% and 35% at 12 and 72 h, respectively. Prolonged retention of bryostatin 1 in mice after i/p injection occurred due to delayed renal excretion, especially during the first 12 h. Considering all pharmacokinetics results, the authors suggested that i/p injection could be a suitable alternative to i/v injection for bryostatin 1 [60].

## 4. Pharmacokinetics Studies in Humans

To date, pharmacokinetic studies in humans have been conducted for very few marine-derived compounds. Most of these molecules are in different stages of clinical trials or are approved for medicinal use. Among others, the anticancer compounds are most promising. The pharmacokinetics publications about anticancer marine-derived molecules are predominant. Most of these investigations have been a continuation of pharmacokinetic experiments in animals.

As a continuation of pre-clinical studies of aplidine and its pharmacokinetics in vivo, several pharmacokinetic studies have been conducted in subjects with advanced tumors. Aplidine (**18**) was administered in infusions (0.13–8.00 mg/m^2^) by different schemes (1 or 24 h weekly; 3 or 24 h biweekly; 1 h for 5 consecutive days every 3 weeks). The group of 48 patients with solid tumor or non-Hodgkin’s lymphoma was enrolled in the phase I of clinical trials. The relatively long T_1/2_ (up to 43.8 h), prolonged MRT (up to 51 h), Cl (up to 6.2 L/h), and high V_ss_ in the blood (up to 144.6 L) were observed in subjects after intravenous infusion of aplidine at the doses of 0.2–8 mg/m^2^ for 24 h. The aplidine concentration in plasma was lower compared to that in blood. The AUC and C_max_ increased with increasing dose. The unchanged drug is excreted with urine (average recovery was ~15% over 48 h) [157]. Similar pharmacokinetic data were later reported by Van Andel et al. [158] who performed experiments with radiolabeled ^14^C aplidine on six patients. Clinical data confirmed previous results observed in animals that aplidine is accumulated in red blood cells. The accumulation was linear up to doses of 5 mg/m^2^. The drug undergoes moderate microsomal-mediated metabolism, [159]. Based on these results, the recommended doses of aplidine were suggested as 5 and 7 mg/m^2^.

During phase I of clinical trials, the pharmacokinetics of dolastatin 10 (**20**) was evaluated on a cancer patient after a 30 s bolus i/v injection at a dose of 65 μg/m^2^. The maximal concentration of dolastatin 10 (29.3 ng/mL) was detected 5 min after injection. The plasma concentration–time function was three-exponential with a t_1/2 α_ of 0.087 h, t_1/2 β_ of 0.69 h, and t_1/2 γ_ of 8 h. The plasma clearance was 3.7 L/h/m^2^ and V_ss_ was 46.7 L/m^2^. The metabolite N-dimethyl dolastatin 10 with m/z 772 was detected in the plasma from 0.75 to 1.5 h. The concentration of metabolite was approximately 1/50th the concentration of dolastatin 10 [153].

The pharmacokinetic data acquired for kahalalide F (**19**) in animal experiments were applied in phase I clinical trials. A group of 32 subjects with advanced androgen refractory prostate cancer received an intravenous injection of infusion (20–930 μg/m^2^) over one hour during five consecutive days every three weeks. The human pharmacokinetics patterns were similar to those observed in animals. The maximal concentration of drug was registered in blood at the end of infusion (C_max_ was 37.9–183.7 ng/mL, AUC was 28.0–53.4 ng/mL) and characterized by dose linearity up to 560 μg/m^2^ per day. Drug elimination was rapid (T_1/2_ = 0.47–0.88 h, Cl = 14.5–6.9 L/h) and was dose dependent; the mean V_ss_ was 7.0 L. Similar to the in vivo results, kahalalide F did not accumulate during the course. The sensitivity of the analytical method was unable to determine the urinary excretion of the drug. Considering these data and based on toxicology, the rational dose of kahalalide F was determined to be 560 μg/m^2^/day [160]. Pardo et al. investigated the pharmacokinetics of kahalalide F in 35 patients with advanced solid tumors after intravenous injection once-weekly as a 1 h infusion [161]. Similar to previous research, linear kinetics for C_max_ and AUC, as well as narrow V_ss_ (5.55 L) and a short T_1/2_ (0.52 h), were observed. The increases in Cl and V_ss_ were strongly correlated with body size and were best predicted with body surface area and height. In a subsequent study, the pharmacokinetics of kahalalide F was reported from a phase II study in patients with advanced malignant melanoma. Patients were infused weakly for one hour i/v with kahalalide F at a dose of 650 μg/m^2^. The median V_ss_ was 7.0 L, and median T_1/2_ was 0.46 h [162]. 

The pharmacokinetics of an antineoplastic alkaloid ecteinascidin 743 (syn. trabectedin) (**25**) isolated from *Ecteinascidia turbinate* (Caribbean sea squirt) was studied in patients with solid tumors. Patients were administered multiple doses (50–900 μg/m^2^) of ecteinascidin 743 by 24 h i/v infusion. The C_max_ (0.06–0.95 ng/mL) and AUC (1.2–36 ng·h/mL) increased linearly with increasing dose. Dose-normalized AUC was independent of dose. This suggests the linear pharmacokinetics of ecteinascidin 743. The pharmacokinetic was also linear after 21 days of five repeated courses. The T_1/2_ increased up to a dose of 400 μg/m^2^, and then decreased. An increase in the clearance after five consecutive courses was registered. At doses of 50–100 μg/m^2^, the concentration of drugs at the terminal time point was under the limit of quantification. The hepatic toxicity of drugs increased with AUC. All data were characterized by considerable interpatient variability. Considering all results together, the authors suggested that a 24 h i/v every 3 weeks is a well-tolerated and appropriate course of treatment [163]. 

The pharmacokinetics of a antineoplastic alkaloid trabectedin (syn. ecteinascidin 743) (**25**) was studied in patients after continuous 24 h intravenous infusion of the drug at a dose of 1.3 mg/m^2^. The maximal concentration (0.6 ng/mL) was observed at 8 h and was maintained at this level until 24 h after injection. The AUC was 16.5 ng·h/mL; Cl and T_1/2_ were 51.3 L/h/m^2^ and 31.9 h, respectively [164]. The results of this experiment allowed recommending a treatment scheme for patients with liposarcoma. In another trial, trabectedin (ecteinascidin 743) was injected by infusion over 1 or 3 h every 21 days to 44 patients with solid tumors. The clearance of trabectedin was not linear over the course of the 1 h infusion schedule. It was not significantly dependent on the dose with the 3 h infusion schedule and did not vary significantly. The AUC showed a linear increase with the dose. The observed hepatotoxicity was significantly associated with higher C_max_ values. The results of this trial suggested a 1650 μg/m^2^ dose of trabectedin for 3 h infusion [165].

Zakirova et al. reported the results of pharmacokinetic studies of histochrome (**17**). Histochrome (1%; 100 mg) was injected intravenously in the seven subjects. The pharmacokinetic data indicated that histochrome has a relatively high AUC (665.6 μg·h/mL), a high volume distribution in plasma (5.7 L), and low clearance (0.16 L/h). The first maximum of the drug in plasma was followed by a second one after 26 h. The authors stated that enterohepatic recirculation over the 2–6 h period leads to prolongation of the elimination half-life (T_1/2_) up to 87.3 h and MRT up to 131.4 h. Presumably, the substance accumulates in adipose tissue, which may explain the prolongation of T_1/2_ and MRT. The study confirmed the rationality of the therapeutic dose selection [166].

A large number of experiments have been performed with fucoidans (**1**). The studies on animals confirmed that high-molecular-weight fucoidan is absorbed after peroral and transdermal application and distributed in tissues and organs [27,28]. It was long believed that fucoidan, due to its high molecular weight and the absence of digesting enzymes in humans, cannot penetrate the blood after peroral administration [167]. Irhimeh et al. detected fucoidan in human blood for the first time after peroral administration. The group of 40 volunteers was administered 3 g of 75% sulfated galactofucan with an MW of 713 kDa isolated from *Undaria pinnatifida* three times a day for 12 days. The median concentration of fucoidan in the plasma was 12.989 mg/L, and the oral bioavailability was calculated as less than 0.6% [31]. In another experiment, 10 healthy male subjects were administered 1 g of fucoidan from *Cladosiphon okamuranus*. Fucoidan was found in serum and urine 3 h after administration. During 9 h of observation, the time-dependent elevations of fucoidan concentration in the serum (up to 100 ng/mL) and urine (up to 1000 ng/mL) were registered. No difference in fucoidan concentration in the serum and plasma was detected. The MW (66 kDa) of the ingested fucoidan in the serum remained unchanged, whereas the MW (1.8–3.1 kDa) of fucoidan in urine was significantly lower, which evidenced the polysaccharide metabolism [49]. Later, a group of 48 volunteers was involved in new experiments. Both male and female subjects were administered 100 g of 100 g of Okinawa mozuku from *Cladosiphon okamuranus* containing 1 g of fucoidan. The highest fucoidan content (37.2 ng/mL in men and 14 ng/mL in women) was observed in urine 9 h after administration [168]. A large study on 396 volunteers was performed with mozuku fucoidan isolated from *Nemacystus decipiens* seaweed. Subjects drank a beverage with 3 g fucoidan [169]. Fucoidan was observed in the urine of 295 subjects. The concentration of fucoidan in urine (as evidenced by ELISA) ranged between 332.3 ± 357.6 and 240.1 ± 302.4 µg/g Cr. Higher concentrations were observed in subjects who consumed more seaweed in their daily diet. The authors confirmed that fucoidan is absorbed after peroral administration in humans and excreted with urine. Unfortunately, the authors did not provide details about fucoidan’s molecular weight, sulfate content, or monosaccharides composition. To understand the effect of *Helicobacter pylori* on fucoidan absorption, the group of 259 subjects consumed 3 g mozuku fucoidan in a health drink [170]. Fucoidan was analyzed in urine. Suppressed absorption of fucoidan was observed in *H. pylori*-positive volunteers aged ≥ 40 years who consumed mozuku once monthly. However, there was no association in the group of *H. pylori*-negative subjects irrespective of the frequency of mozuku consumption and age. Notably, the basal fucoidan level in urine was significantly higher in the *H. pylori*-positive group. This evidenced the contribution of *H. pylori* to fucoidan metabolism. Taken together, these results showed that fucoidan is absorbed after peroral administration and metabolized in the human excretory system. However, its concentration in blood and urine was relatively low [171]. This is in agreement with in vivo data that evidenced the rapid absorption and slow elimination of fucoidan after peroral administration [27,28,35]. We have not found publications that reported the other pharmacokinetic parameters of fucoidan, such as AUC, Cl, T_1/2_, etc., in humans. Such studies are essential for better understanding the mechanisms of fucoidan efficacy.

The bioavailability and pharmacokinetics of the synthetically derived astaxanthin *E/Z*-isomer were demonstrated in three healthy male volunteers. Subjects were treated with water-dispersible astaxanthin containing beadlets at a single dose of 100 mg. Astaxanthin was found in plasma, and no metabolites were detected by HPLC. The C_max_ of 1.3 mg/L was observed at 6.7 ± 1.2 h. The AUC was 42 ± 3 mg·h/L. The blood circulation was prolonged. T_1/2_ varied between 11.4 and 32.1 h depending on the subject. About 12% of the maximal concentration of astaxanthin was detected in plasma 72 h after administration. Astaxanthin was presented in high- and low-density plasma lipoproteins with a prevalence of very low-density lipoproteins containing chylomicrons [172]. The pharmacokinetics of astaxanthin was studied in eight healthy male volunteers after a single dose administration of commercially available capsules filled with the spray-dried cells of microalga *Haematococcus pluvialis* (40 mg of astaxanthin per capsule). The maximal concentration in plasma (C_max_ = 55.2 ± 15.0 μg/L) was achieved in all subjects 8 h after administration, and the AUC was 1347 ± 501 μg·h/L. Astaxanthin relatively prolonged time was circulated in the blood (T_1/2_ = 16.7 ± 7.2 h) [173]. Results about the bioavailability and pharmacokinetics of astaxanthin were published by Okada et al. Commercially available *Haematococcus* algal extract containing 48 mg of astaxanthin was consumed by smoking and non-smoking volunteers. The bioavailability of astaxanthin was higher when administered after a meal than before a meal (AUC = 7526 vs. 2996 μg·h/L). The meal delayed absorption (T_max_ after a meal was reached approximately three-fold later than before a meal) and lowered clearance approximately by two-fold. The difference in MRT was not statistically significant. Smoking reduced the half-life elimination time of astaxanthin. The authors suggested that fats in the meal stimulate excretion of bile, which helps the dispersion of this carotenoid and results in more effective absorption [174]. This hypothesis is in line with the previously published data, which demonstrated the significant increase in astaxanthin bioavailability in the formulation of astaxanthin with fats [173].

We have not found the data about chitosan pharmacokinetics in the literature. However, the number of published randomized controlled clinical trials indicates that dietary supplements are useful for weight control due to the ability of chitosan to bind fats in the gastrointestinal tract, followed by its excretion with feces [90].

## 5. Concluding Remarks

The intensive study of marine organisms in recent years has led to the isolation and identification of an enormous number of new compounds. Further screening resulted in the selection of the most fascinating and potent candidates, which have entered extensive pre-clinical and clinical trials. However, insufficient attention has been paid to pharmacokinetics. In this review, we summarized and examined the data on the pharmacokinetics of marine-derived molecules with a focus on their absorption, distribution, metabolism, and excretion in animals and humans. Most often, data on the pharmacokinetics of marine drugs are fragmentary. Not all significant parameters have been calculated and reported in articles. In general, pharmacokinetics is studied after a single dose administration of the molecule. The data on pharmacokinetics after repeated dose administration or after different doses’ administration have very rarely been published. These data have a considerable impact on dose selection for clinical study design.

Pharmacokinetics data are essential for formulation development [175]. The study of absorption provides insight into the proper administration route. If a drug candidate has a high absorption rate, it could be used for formulation as such, or modified for delayed/controlled release. For poorly absorbed candidates, nanoformulation, use of solubilizers, conjugation with external transporters, or other technological modification is required. The understanding of the pharmacokinetics of marine-derived polysaccharides (alginates chitosan, fucoidan) has led to their extensive use not only as drugs but also for improving the bioavailability of poorly soluble compounds in pharmaceutical formulations [176,177].

The distribution of compounds in organs and tissues has seldom been studied. Such experiments require additional resources. The activity of a compound is associated with its distribution in tissues/organs; therefore, the investigation of distribution is helpful for interpreting pharmacological activity results and allows the determination of possible toxic effects with intensive drug accumulation in particular organs. The lighting of the tissues in which drugs accumulate allows the prediction of new targets and activities. The marine-derived drugs are known for their activity at low doses. Therefore, sometimes not all pharmacokinetic parameters have been determined due to the sensitivity of analytical techniques. The biomarker approach could be helpful for drug candidates that have a structure similar to endogenous molecules. This approach could assist with the identification of the organ/tissue in which the drug is metabolized. However, it does not provide information about drug metabolites. We know a little about drug metabolism. Often, metabolites exhibit more prominent pharmacological activity than the parent substances. Insufficient attention has been paid to metabolism in pharmacokinetics studies. Understanding metabolism reveals new possibilities for the evaluation of mechanisms of action and the development of highly effective drugs. Scientific reports with systematized information on the distribution of drugs in organs and tissues, metabolism, and excretion are highly anticipated. Consecutive investigations of promising drug candidates confirmed that the correct pharmacokinetics results obtained on animals are essential for smooth translation into phase I clinical trials.

The efforts toward the development of marine-derived therapeutics are steadily opening up new perspectives. The in-depth characterization of pharmacokinetics can generate information to help us understand the molecular basis behind the pharmacological activity, correct doses and treatment schemes selection, and result in more effective drug application. Thus, an increase in pharmacokinetics research of marine-derived compounds is expected in the near future. 

## Figures and Tables

**Table 1:** Analytical methods used for the analysis of marine-derived compounds in biomaterials.

Method	Recovery (%)	Linearity Range (µg/mL)	Compound	Source	Biomaterial	Reference
Biomarker assay Anti-Xa activity	-	0.027–0.217	Fucoidan	Brown algae *Fucus vesiculosus*	Plasma, Tissue	[27]
Biomarker assay Anti-Xa activity	-	0.014–1.13 *	Fucoidan	Brown algae *Fucus vesiculosus*	Skin Tissue	[28]
Biomarker assay Dypeptydylpeptidase IV inhibition	-	1.9–710	Lipid extract of gonads	Sea urchins *Strongylocentrotus droebachiensis*	Plasma	[29]
Biomarker assay Lactate dehydrogenase activity	-	0.01–7.05	Glycopeptide	Sea urchins *Strongylocentrotus droebachiensis*	Plasma Tissue	[30]
Competitive ELISA	-	0.078–80	Fucoidan	Brown algae *Undaria pinnatifida*	Plasma	[31]
Fluorescent labeling	-	-	Fucoidan	Brown algae *Fucus vesiculosus*	Plasma Organs	[32]
Gas chromatography	82–87	0.3–10	Halomon	Brown algae *Fucus vesiculosus*	Plasma Organs	[33]
HPLC with derivatization	96.6–106.4	0–80 ***	Fucoidan	*Laminaria japonica*	Plasma	[34]
HPLC with fluorescence detection	93.3–96.5	0.5–100	Fucoidan	Laminaria japonica	Plasma	[35]
HPLC with postcolumn fluorescence derivatization	95.5–99.2	0.5–150	Fucoidan	Laminaria japonica	Serum	[36]
HPLC with UV detection	86.2–100.1	0.5–500	Diindolinonepyrane (2,5-BHPA)	Marine fungi *Stachybotrys longispora*	Plasma	[37]
HPLC-ELSD	90.0	0.1–5 0.1–20	Echinoside A Holotoxin A1	Sea cucumber *Apostichopus japonicus*	Plasma	[38]
HPLC-ESI-MS	85.0	0.3–20 0.2–10	Echinoside A holothurin A	Sea cucumbers *Pearsonothuria graeffei*	Serum, liver	[39]
HPLC-ESI-MS	96.6–102.3	0.5–500	2,5-BHPA	Marine fungi *Stachybotrys longispora*	Plasma	[40]
LC-MS/MS	88.0	0.025–0.25	Frondoside A	Sea cucumber *Cucumaria frondosa*	Plasma	[41]
LC-MS/MS	92.9–101 84.7–91.6	0.00153–0.72 0.00117–0.6	Fucoxanthin Fucoxanthinol		Plasma	[42]
LC-MS/MS	80.0	1.0–100	Sodium alginate	-	Plasma, urine	[43]
LC-MS/MS	69 91	0.005–0.1 0.00125–0.125	Aplidine (dehydrodidemnin B)	Mediterranean tunicate *Aplidium albicans*	Plasma, urine	[44]
LC-MS/MS	90–101 90–94	0.01–5 ** 0.5–50 **	Sesterterpene MHO7 (6-epi-ophiobolin G)	Mangrove fungus *Aspergillus ustus*	Plasma tissues	[45]
LC-MS/MS	-	0.002–0.8	Ilimaquinone Epimers	Marine Sponge *Hippiospongia metachromia*	Plasma	[46]
MALDI-MS	-	0.001–1.000	Cucumarioside A_2_-2	Sea cucumber Cucumaria japonica	Tissue	[47]
RIA	-	10 pg–10 ng	Didemnin B	Caribbean tunicate (sea squirt) *Trididemnum solidum*	Plasma	[48]
Sandwich ELISA	97–105 86–113 97–98	0.001–0.1	Fucoidan	Brown algae *Cladosiphon okamuranus*	Serum, plasma, urine	[49]
TRA	77.4	-	Aplidin (Plitidepsin)	-	Urine	[48]

* Linearity range in mg/g, ** linearity range in μM/L, and *** linearity range by fucose. –, no data provided by authors, TRA, total radioactivity analysis; RIA, radioimmunoassay; MALDI, matrix-assisted laser desorption/ionization; IMS, MALDI imaging mass spectrometry; HPLC, high-performance liquid chromatography; LC, liquid chromatography; ELSD, evaporative light-scattering detection; MS, mass spectrometry; 2,5-BHPA, 2,5-bis-[8-(4,8-dimethyl-nona-3,7-dienyl)-5,7-dihydroxy-8-methyl-3-keto-1,2,7,8-tertahydro-6H-pyran[a]isoindol-2-yl]-pentanoic acid.

**Table 2:** The main pharmacokinetic parameters of some marine-derived compounds in plasma.

Compounds	Animals/Dose (mg/kg/vehicle)	Administration	T_max_ (h)	T_1/2_ (h)	C_max_ (µg/mL)	AUC_0−t_ (µg·h/mL)	Reference
Fucoidan (**1**) MW 735 kDa	Rats/100/ointment	i/v topical	N.d.1.0	9.5 20.7	9.2 0.15	10.8 1.9	[28]
Fucoidan (**1**) MW 735 kDa	Rats/100/starch slime	peroral	3.2	3.4	0.13	0.99	[27]
Fucoidan (**1**) MW 107.8 kDa	Mice/50/phosphate buffer solution (pH 7.4)	i/v	0.5	2.77	66.4	138.7	[32]
Griffithsin	Rats/10/phosphate-buffered saline (pH 7.4)	i/v s/c	- 4.0	10.7 13.8	81.8 6.6	0.11 0.045	[51]
Sodium alginate (**2**)	Mice/10 mg/500 µL saline	peroral	0.08	N.d.	24.5	N.d.	[43]
Halomon (**3**)	Mice/135/cremophor–EtOH–0.154 M NaCl (1:1:6, by vol.)	i/v	N.d.	8.4	N.d.	189,960	[33]
		i/p	N.d.	12.3	N.d.	85,620	
		s/c	N.d.	8.0	N.d.	89,280	
		peroral	N.d.	4.5	N.d.	7080	
Fucoxanthin (**5**)	Rats/2/mixed micelle	i/v	N.d.	2.3	N.d.	9.86	[42]
Fucoxanthinol (**23**)			1.0	11.9	0.59	3.26	
Fucoxanthin (**5**)	Rats/65/mixed micelle	peroral	7.7	1.2	0.03	0.19	[42]
Fucoxanthinol (**23**)			11.0	N.d.	0.26	5.0	
Astaxanthin (**6**)	Rats/10/polyethylene glycol 400–*N*,*N*-dimethylacetamide 50:50, *v*/*v* Rats/100/the same solution	i/v peroral	N.d. 6.5	N.d. N.d.	50 0.08	29,280 4638	[52]
Chitosan (**7**) lactate	Rats/20/phosphate buffered saline pH 7.4	peroral					[53]
MW 3.8 kDa			0.5	N.d.	20.23	24.13	
MW 7.5 kDa			0.5	N.d.	9.30	11.55	
MW 13 kDa			0.5	N.d.	5.86	8.71	
MW 22 kDa			0.5	N.d.	4.32	5.59	
MW 230 kDa			0.5	N.d.	<0.5	0.97	
Chitosan (**7**)	Mice/500/1% (*v*/*v*) acetic acid solution	peroral					[54]
MW 0.99 kDa			0.5	N.d.	680	N.d.	
MW 39.1 kDa			1.0	N.d.	190	N.d.	
MW 32.7 kDa			1.0	N.d.	310	N.d.	
MW 760 kDa			0.5	N.d.	60	N.d.	
Saponin extract Echinoside A (**8**) Holothurin A (**9**)	Rats/30/0.9% saline	peroral		N.d.		N.d.	[39]
			3.0		0.83		
			7.0		0.24		
			3.0		0.34		
			9.0		0.27		
Echinoside A (**8**) Holotoxin A1 (**10**)	Rats/20/water	peroral	3.0	6.9	0.9	9.3	[38]
		i/v	0.08	8.5	4.0	16.4	
		i/v	0.08	4.4	2.9	6.5	
Frondoside * A (**11**)	Mice/0.1/0.7% DMSO in saline	i/v	0.08	8.5	0.17	0.73	[41]
		i/p	1.0	14.0	0.024	0.16	
Cucumarioside * A_2_-2 (**12**)	Mice/5/water	i/p	0.64	15.1	62.6	1544	[55]
		peroral	0.67	0.35	74.4	1680	
MHO7 (6-epi-ophiobolin G) (**13**)	Mice/500/corn oil	peroral	8.0	6.97	1.38	10.50	[45]
2,5-BHPA ** (**14**)	Rats/20/normal saline with NaHCO_3_	i/v	N.d.	N.d. 23.2	N.d.	53,940	[37]
2,5-BHPA (**14**)	Dogs/7.5/normal saline with NaHCO_3_	i/v	N.d.	0.82	56.5	19.7	[40]
Mixture Ilimaquinone (**15**) + epi-Ilimaquinone	Rats/2 + 1/corn oil	i/v	N.d.	0.6 0.4	N.d.	1.46 0.24	[46]
Mixture Ilimaquinone (**15**) + epi-Ilimaquinone	Rats/20 + 10/corn oil	peroral	1.3 1.7	3.8 3.9	1.3 0.1	5.5 0.3	[46]
Manzamine A (**16**)	Rats/10/EtOHRats/50/water	i/v peroral	N.d.	N.d.	40	N.d.	[56]
			10	N.d.	1.1	N.d.	
Glycopeptide	Rats/0.1/water	i/v i/n	N.d.	0.80	6.15	8.00	[30]
	0.05/water		0.67	0.77	2.90	3.93	
	0.1/water		0.75	3.53	4.15	7.14	
	0.2/water		0.70	4.03	6.22	12.6	
Lipid extract of gonads	Rabbits/15/starch slime	peroral	3.0	8.8	107	313	[29]
Aplidine (**18**) (dehydrodidemnin B)	Rats/0.7/EtOH, cremophor EL 10% in saline	i/v	N.d.	0.15	0.1	N.d.	[44]
Kahalalide F ** (**19**)	Mice/0.278/dimethylformamide/sterile saline 10:90 (*v*/*v*)	i/v	N.d.	0.26 4.4	0.001	N.d.	[57]
Dolastatin 10 (**20**)	Mice/0.24/water Mice/0.32/water	i/v	N.d.	5.6	0.28	0.067	[58]
		i/p		N.d.	N.d.	0.011	
		s/c		3.7	0.011	0.058	
Dolastatin 10 ** (**20**) Dolastatin 15 ** (**21**)	Mice/1/water Mice/1/water	i/v i/v	N.d. N.d.	0.04 1.6 0.09 0.52	N.d. N.d.	0.33 0.21	[59]
Bryostatin 1 ** (**22**)	Mice/0.04/phosphate buffer containing 30% DMSO	i/v i/p	N.d. N.d.	1.05 22.96 0.81 28.76	0.092 0.013	0.37 0.62	[60]

Note: i/v, intravenous; i/p, intraperitoneal; s/c, subcutaneous; i/n, intranasal; T_1/2_, apparent half-life of elimination; AUC_0–t_, the area under the curve; C_max_, maximum concentration (μg/mL) for plasma; T_max_, time at which C_max_ is observed. MRT, mean residence time; the results are expressed as the mean ± SD; *t*_1/2_ α, distribution half-life; *t*_1/2_ β, elimination half-life. * mean values (*n* = 5); ** $\frac{t1/2\;\alpha }{t1/2\;\beta }$ (for two-compartment model); N.d., no data; MW, molecular weight.

**Table 3:** Tissue distribution and retention of marine-derived compounds after administration.

Compound	Animals/Dose (mg/kg/administration)	T_max_ (h)							C_max_ (µg/g)							Reference
		Heart	Stomach	Liver	Spleen	Lung	Kidney	Brain	Heart	Stomach	Liver	Spleen	Lung	Kidney	Brain	
Fucoidan (**1**) MW 735 kDa	Rats/100/peroral	N.d.	N.d.	2	3	N.d.	5	N.d.	N.d.	N.d.	0.53	0.78	N.d.	1.23	N.d.	[27]
Fucoidan (**1**) MW 107.8 kDa	Mice/50/i/v	N.d.	N.d.	0.5	6	4	0.5	N.d.	N.d.	N.d.	284	78	111	1092	N.d.	[32]
Griffithsin **	Mice/50/s/c	N.d.	N.d.	N.d.	N.d.	N.d.	N.d.	N.d.	-	-	2.5	6.0	-	4.6	-	[68]
Fucoxanthin (**5**) Fucoxanthinol (**23**) Amarouciaxanthin A (**24**)	Mice/0.105 mg per 200 µL/peroral	4 4	N.d. N.d.	4 4	4 4	4 4	4 4	N.d. N.d.	0.15 0.069	N.d. N.d.	0.38 0.12	0.16 0.063	0.28 0.12	0.15 0.067	N.d. N.d.	[80]
Astaxanthin (**6**)	Rats/100/peroral	8	8	8	8	8	8	8	0.12	7.3	0.14	0.16	0.15	0.21	0.26	[52]
Cucumarioside * A_2_-2 (**12**)	Mice/5/i/p	0.42	0.05	0.067	0.42	N.d.	0.067	N.d.	120	158	69	69	N.d.	73	N.d.	[55]
	Mice/5/peroral	0.33	0.17	0.33	0.33	N.d.	0.33	N.d.	95	153	74	52	N.d.	70	N.d.	
MHO7 (6-epi-ophiobolin G) (**13**)	Mice/50 mg/kg/peroral	4	8	1	8	12	12	1	0.95	8.4	4.0	0.65	2.5	8.0	1.0	[45]
2,5-BHPA (**14**)	Rats/20/i/v	0.25	0.25	0.25	0.25	0.25	0.25	N.d.	2.4	4.1	235	16.8	16.0	18.0	-	[37]
2,5-BHPA (**14**)	Dogs/7.5/i/v	1	N.d.	1	1	1	1	1	1.5	N.d.	52	5.0	6.0	7.5	3.5	[40]
Glycopeptide	Rats/0.1/i/n	N.d.	N.d.	1.6	2.4	N.d.	3.6	N.d.	N.d.	N.d.	0.73	2.53	N.d.	0.98	N.d.	[30]
Bryostatin 1 (**22**)	Mice/0.04/i/v	1	1	0.5	1	0.5	1	0.5	0.04	0.027	0.900	0.060	1.0	0.050	0.002	[60]

* mean values (*n* = 5); ** samples from different tissues were collected after each experiment. N.d., no data; MW, molecular weight; -, below the limit of detection.
